# Biomarkers in Metastatic Colorectal Cancer: Status Quo and Future Perspective

**DOI:** 10.3390/cancers14194828

**Published:** 2022-10-03

**Authors:** Alberto Puccini, Andreas Seeber, Martin D. Berger

**Affiliations:** 1 Medical Oncology Unit 1, IRCCS Ospedale Policlinico San Martino, 16132 Genoa, Italy; 2 Department of Internal Medicine and Medical Specialties (DIMI), School of Medicine, University of Genoa, 16132 Genoa, Italy; 3 Department of Hematology and Oncology, Comprehensive Cancer Center Innsbruck, Medical University of Innsbruck, 6020 Innsbruck, Austria; 4 Department of Medical Oncology, Inselspital, Bern University Hospital, University of Bern, 3010 Bern, Switzerland

**Keywords:** colorectal cancer, metastatic, biomarker, precision medicine, personalized treatment

## Abstract

**Simple Summary:**

Despite recent progress in the treatment of metastatic colorectal cancer patients, novel therapeutic approaches are highly warranted to further improve outcome. Additionally, it is of utmost importance to define subgroups of patients who may derive the most benefit from a specific treatment, as we are moving forward in the field of personalized medicine. This review provides an overview about established biomarkers helping us to better guide treatment. Moreover, we report on emerging and potential promising biomarkers having the potential to be implemented into clinical practice in the near future.

**Abstract:**

Colorectal cancer (CRC) is the third most frequent cancer worldwide, and its incidence is steadily increasing. During the last two decades, a tremendous improvement in outcome has been achieved, mainly due to the introduction of novel drugs, targeted treatment, immune checkpoint inhibitors (CPIs) and biomarker-driven patient selection. Moreover, progress in molecular diagnostics but also improvement in surgical techniques and local ablative treatments significantly contributed to this success. However, novel therapeutic approaches are needed to further improve outcome in patients diagnosed with metastatic CRC. Besides the established biomarkers for mCRC, such as microsatellite instability (MSI) or mismatch repair deficiency (dMMR), *RAS/BRAF*, sidedness and *HER2* amplification, new biomarkers have to be identified to better select patients who derive the most benefit from a specific treatment. In this review, we provide an overview about therapeutic relevant and established biomarkers but also shed light on potential promising markers that may help us to better tailor therapy to the individual mCRC patient in the near future.

## 1. Introduction

Colorectal cancer (CRC) is a leading cause of cancer-related mortality worldwide, and its global incidence is continuously increasing [1]. Thus, the global burden of CRC will pose significant challenges to the health care system due to increasing financial costs [2]. Whereas the median overall survival (mOS) of patients with metastatic CRC (mCRC) did not exceed 6 months in the eighties, nowadays the median survival reaches almost 30 months [3,4]. The implementation of upfront antiangiogenic agents in right-sided *RAS* wild-type mCRC, the addition of anti-epithelial growth factor receptor (EGFR) antibodies in left-sided *RAS* wild-type mCRC, and the introduction of tyrosine kinase inhibitors and further chemotherapeutics have led to a significant improvement in prognosis over time [5,6,7,8]. Certainly, the establishment of interdisciplinary tumor boards, advances in surgical techniques and local ablative and stereotactic procedures, especially in oligometastatic disease, have further contributed to this successful development [9]. Additionally, CPIs have significantly improved OS in approximately 3–5% of mCRC patients harboring microsatellite instable (MSI) or mismatch repair-deficient (dMMR) tumors [10]. Patients harboring a *BRAF*^V600E^ mutation (8–15%) undergoing a combination therapy with encorafenib and cetuximab in second- and further-lines have demonstrated a longer survival compared to those who received physician’s choice therapy [11]. Currently, the efficacy of this combination is further explored in the first-line setting [12]. Moreover, just recently, several small clinical phase 1 and 2 trials demonstrated encouraging response rates in pretreated mCRC patients harboring *KRAS*^G12C^ mutations (3–4% of all mCRC), *NTRK*- (0.5%) or *RET* fusions (0.2–1.6%) who underwent treatment with *KRAS*^G12C^ inhibitors in combination with anti-EGFR agents, larotrectinib or entrectinib, or selpercatinib, respectively [13,14,15,16]. However, advances in drug development are highly warranted to further improve prognosis. In the past, research focused more towards directly targeting the tumor cells, while neglecting the crucial role of the tumor microenvironment (TME) and its influence on tumor progression. A growing body of evidence nowadays indicates that targeting cells within the TME might be promising to further enhance anti-tumorigenic properties or to impede pro-tumorigenic effects [17,18]. To keep up with the latest progress in drug development, further biomarkers have to be defined and promising platforms such as ex vivo culturing of tumors are to be established to help personalize treatment and to identify patients who may benefit most from a specific therapy. In the present review, we will provide an overview about currently established biomarkers that guide clinical decision-making but also shed light on promising markers that may be clinically useful in the near future. Table 1 outlines a selection of requirements for an effective prognostic or predictive biomarker [19,20,21,22,23]. While a prognostic biomarker predicts the outcome of a disease irrespective of the applied treatment and thus may serve as an independent indicator of tumor biology, a predictive biomarker predicts the outcome of a disease depending on a specific treatment [24]. Diagnostic biomarkers do not play a role in mCRC. An elevation of the carcinoembryonic antigen (CEA) can indicate or support the presence of colon cancer but is not diagnostic, as an increased CEA level is rather unspecific and can also be observed in the presence of inflammation or other malignancies. Conversely, a normal CEA level does not exclude colon cancer.

## 2. Established Biomarkers in mCRC

The advances of precision medicine have produced unprecedented paradigm shifts in the treatment algorithms of advanced cancers. However, CRC remains far behind the other solid tumors (e.g., non-small cell lung cancer, melanoma, genitourinary tract cancers). To date, only a few biomarkers have sufficient actionable and clinical implications to warrant international guideline-recommended routine testing [25]. Table 2 provides an overview of established biomarkers in mCRC.

Only in approximately 5% of mCRC patients, namely those harboring an MSI/dMMR tumor, we observed outstanding and practice-changing results with CPI therapy. The treatment of the remaining vast majority of mCRC patients (who are microsatellite stable—MSS) is still based upon only a few molecular and/or clinical determinants: sidedness of the primary tumor location, *RAS* and *BRAF* mutational status [26]. Another well-established biomarker is *HER2* amplification, since several trials have shown the clinical activity of a combination strategy for HER2 blockade [27], although no standard treatments have been approved by regulatory agencies for these patients.

*NTRK* fusions are extremely rare in CRC patients (<1%). Despite this, the recent positive and practice-changing basket trials of TRK inhibitors (larotrectinib [28] and entrectinib [15]) encourage clinicians to test for *NTRK* in mCRC patients, especially in later-lines of therapy [29]. Several other molecular determinants are under clinical investigation. Some of them are expected to become part of our practice routine and change our therapeutic paradigms in the near future: circulating tumor DNA (ctDNA), *POLE* alteration, *KRAS*^G12C^ mutation, *FGFR* fusions, altered DNA damage repair (DDR) genes, tumor mutational burden (TMB) and consensus molecular subtypes classification (CMS), as well as *ALK/RET/ROS* fusions [30].

### 2.1. Sidedness

Primary tumor sidedness (right- versus left-sided) is considered an established and crucial prognostic and predictive factor in the treatment of mCRC [31,32,33,34]. Besides the difference in their origin, these tumors exhibit different histology. While right-sided tumors are often associated with sessile serrated adenomas or mucinous adenocarcinomas, left-sided tumors are related to tubular, villous and typical adenocarcinomas. Usually, left-sided tumors exhibit a polypoid growth pattern and are therefore easier to detect compared to right-sided tumors which are more frequently flat [35].

Comparative molecular analyses of right- and left-sided CRCs revealed molecular distinctions, such as different mutation rates in *TP53, KRAS*, *PIK3CA*, *BRAF* and diverse methylation patterns, as well as different MSI/dMMR rates [36,37,38]. Another study demonstrated that right-sided colon cancers had higher rates of MSI, more frequent aberrant activation of the EGFR/MAPK pathway including higher *BRAF* mutation rates and an increased mutational burden compared to left-sided CRCs [36].

A retrospective study using data from the SEER registry demonstrated that patients with right-sided stage III or IV colon cancers had worse survival outcomes in comparison to left-sided CRC patients [39]. Another study indicated that right-sided colon cancers were associated with a high CpG island methylator phenotype (CIMP) and *BRAF* mutations, translating into inferior OS and a worse response to anti-EGFR treatment [40]. 

Therefore, one may assume that mCRC with primary tumors located in the left colon display a different tumor biology with a different response to therapy compared to those arising in the right colon. This hypothesis has been confirmed in multiple exploratory post-hoc analyses of large, randomized trials [8,33,41,42,43] (see Table 2). Just recently, the findings of the phase III PARADIGM study have been presented at the ASCO Annual Meeting 2022. Patients with left-sided *RAS* wild-type mCRC treated with FOLFOX and panitumumab showed a prolonged mOS compared to those receiving FOLFOX and bevacizumab in the first-line setting (37.9 vs. 34.3 months, HR 0.82, *p* = 0.031) [44,45]. 

For these reasons, to date, the treatment algorithm for mCRC patients in the first-line setting is based on the primary tumor location: chemotherapy plus anti-EGFR regimen is recommended for left-sided MSS, *RAS* and *BRAF* wild-type tumors, while chemotherapy plus anti-VEGF (bevacizumab) is recommended for right-sided MSS mCRC patients [46]. 

### 2.2. DNA Mismatch Repair Deficiency (dMMR) and/or Microsatellite Instability (MSI)

dMMR comprises about 15% of all CRC. It is more frequently observed in early- than in late-stage disease (stage I–II: 15–20%, stage III: 10–15%, stage IV: around 5%) [47,48]. The mismatch repair system (MMR) status can be investigated by testing for loss of an MMR protein by immunohistochemistry (IHC) or for MSI using a PCR- or NGS-based assay. dMMR or MSI-high (MSI-H) tumors display the loss of at least one MMR protein with IHC (MLH1, MSH2, MSH6, and PMS2) or instability in two or more of the five microsatellite markers (BAT25, BAT26, D2S123, D5S346 and D17S250) with a PCR-based assay [49,50], respectively. Concordance between these two techniques is quite high (>90%) [51], thus usually IHC testing is performed to determine the MMR status. However, further PCR- or NGS-based testing is recommended in case of undetermined or inconclusive IHC findings [26,52]. MSI-H/dMMR mCRC are more frequently right-sided and poorly differentiated and correlate more often with *BRAF* mutations [49]. 

Testing for dMMR/MSI-H status is mandatory and crucial for the management of CRC patients for mainly four reasons: (a) universal screening for Lynch syndrome, which is the most common cause of hereditary CRC [53,54]; (b) dMMR status is one of the most studied and well-established biomarkers for better prognosis, especially in stage II CRC patients. In all the studies, MSI-H has been associated with a better outcome in comparison to MSS, in terms of time to recurrence (TTR), relapse-free survival (RFS) and OS [55,56]; (c) in stage II, CRC dMMR status is also a strong negative predictive biomarker of 5-fluorouracil efficacy, thus guiding adjuvant chemotherapy choice [57]; (d) in the metastatic setting, MSI-H/-dMMR status is a strong positive factor for predicting response upon CPI, as was first shown by the landmark studies by Le et al. [58,59]. Recently, the final analysis of the phase II KEYNOTE-164 trial was presented, yielding durable and clinical meaningful benefit, with a 30% plateau of both progression-free survival (PFS) and OS curves at five years and a manageable safety profile in patients with previously treated MSI-H/dMMR mCRC [60].

MSI-H/dMMR status is a well-established biomarker predictive of CPI response. The rationale is based on the significantly increased number of mutation-associated neoantigens resulting from mismatch-repair deficiency. In fact, MSI-H mCRC has an immunogenic TME, characterized by dense immune infiltration and a Th1-associated cytokine-rich environment [61]. Several trials demonstrated that CPI as monotherapy or combined with other agents were associated with improved clinical outcome in this subgroup of patients [62,63]. Currently, the phase II CheckMate 142 trial explores the impact of nivolumab monotherapy and in combination with ipilimumab in previously treated or untreated patients with MSI-H/dMMR or MSS/MMR-proficient mCRC [64]. A robust and durable clinical benefit with a manageable safety profile in previously treated patients with MSI-H/dMMR mCRC was shown [62,63]. A recent report with 4 years of follow-up confirmed impressive and unprecedented results with nivolumab (3 mg/kg) plus low-dose (1 mg/kg) ipilimumab in this setting: ORR was 65%, with a disease control rate (DCR) of 81%. The 48-month rates of PFS and OS were 53% and 71%, respectively [64]. 

Based on these results, nivolumab combined with ipilimumab obtained approval in Europe, while Japan and the United States approved nivolumab as a single-agent treatment and combined with ipilimumab in pretreated MSI-H/dMMR mCRC patients. These exceptional findings have also been confirmed in the first-line setting: with a median follow-up of 29.0 months, nivolumab plus low-dose ipilimumab showed an ORR and a DCR of 69% and 84%, respectively. Furthermore, the 24-month rates of PFS and OS were 74% and 79%, respectively [65]. In this setting, the results are awaited from the current phase III CheckMate 8HW trial (NCT04008030).

Finally, the KEYNOTE-177 phase III randomized trial showed that pembrolizumab led to significantly longer PFS than chemotherapy plus antibody (16.5 vs. 8.2 months) in newly diagnosed MSI-H/dMMR mCRC, with significant fewer treatment-related adverse events [10]. Moreover, 3-year OS (3-yr OS) was 61.4% in the experimental arm, much higher than that of the control arm (3-yr OS 50.3%) [66]. Considering these results, the Food and Drug Administration (FDA) first and then the European Medicines Agency (EMA) approved pembrolizumab alone as a first-line treatment for patients with MSI-H/dMMR mCRC.

These results led to a paradigm shift in the management of MSI-H/dMMR mCRC patients in recent years [67]. Many studies are still warranted to improve our knowledge, especially about primary resistance to CPIs, since up to 30–40% of MSI-H/dMMR mCRC patients do not benefit and progress. This may be caused by different reasons, such as the misdiagnosis of the actual dMMR status [68], microenvironment characteristics and the composition of the gut microbiome [69], as well as the presence of ascites and peritoneal involvement [70]. Nevertheless, the benefit for the majority of MSI-H/dMMR mCRC patients treated with CPI is unprecedented, with one-third of patients who could achieve a complete response. This makes MMR status mandatory and extremely useful in clinical practice.

To underscore the revolution in the management of patients with MSI-H/dMMR, it is noteworthy that neoadjuvant CPI also proved to be a promising new approach for the management of MSI-H/dMMR non-metastatic CRC [71,72,73,74].

### 2.3. RAS Mutations

The *RAS* oncogene family consists of three oncogenes in humans, located on chromosome 12, namely Kirsten (*KRAS*), Neuroblastoma (*NRAS*) and Harvey rat sarcoma viral oncogene homolog (*HRAS*). The *RAS* family is one of the most frequently mutated genes across all malignancies, including CRC [75], and functions as regulators of the intracellular signaling transduction cascades involved in cell growth, differentiation and survival [76]. *RAS* stimulates various downstream signaling pathways, including the RAS-RAF-MEK-ERK and the phosphatidylinositol 3-kinase (PI3K) pathways [77].

Approximately 40–50% of CRC carry *KRAS* or *NRAS* mutations. The majority of them are found in *KRAS* G12, G13 or Q61 codons. Of these, G12 variants are the most frequent ones (around 70%) [78]. 

*RAS* (and *BRAF*) mutations confer more aggressive tumor biology and are negative prognostic factors in particular in MSS mCRC. Moreover, these genetic alterations are negative predictive factors for a response to milestone anti-EGFR therapy (cetuximab or panitumumab) [79,80,81]. 

For a long time, *RAS* mutations have been deemed ‘undruggable’ [82], as many attempts targeting *RAS* mutations failed in the past. However, the recent development of *KRAS^G12C^*-selective inhibitors established a foundation for the development of inhibitors suitable for clinical testing and opened new therapeutic strategies for this historically undruggable target [83,84].

Determination of primary tumor location, MMR status and *RAS*/*BRAF* mutational status is mandatory to assess the optimal therapeutic strategy in the first-line setting. As mentioned above, patients with MSS, *RAS* and *BRAF* wild-type mCRC and a primary tumor located in the left colon are considered suitable candidates for first-line anti-EGFR treatment in combination with chemotherapy [4,31,32,33,85,86,87].

Despite this selection of patients, primary resistance to anti-EGFR agents still exists, and more studies are requested to dig deep into the mechanisms of resistance. For these reasons, Cremolini et al. demonstrated a promising negative predictive impact of the PRESSING panel: uncommon molecular alterations linked to primary resistance to EGFR inhibition, including *HER2* amplification/activating mutations, *MET* amplification, *NTRK/ROS1/ALK/RET* rearrangements, *PIK3CA* exon 20, *PTEN* and *AKT1* mutations [88]. This negative molecular hyperselection of patients, added to the initial assessment of sidedness and *RAS/BRAF* mutational status, may help identify a subgroup of patients who will exceptionally benefit from anti-EGFR–based initial therapy [89].

#### *KRAS^G12C^* Mutation

The specific *KRAS^G12C^* mutation is detectable in 1–3% of CRCs [83,90,91,92]. Only recently, two oral inhibitors (sotorasib, adagrasib) demonstrated clinical activity in mCRC patients harboring a *KRAS^G12C^* mutation [93,94]. In a phase I trial, sotorasib yielded a DCR of 73.8% and a PFS of 4.0 months [95]. These results have been confirmed by the subsequent phase II CodeBreak trial (ORR: 9.7%, PFS: 4.0 months) [96]. Furthermore, the KRYSTAL-1 phase I/II trial investigated the clinical activity of adagrasib in pretreated mCRCs. These promising findings revealed an ORR of 22%, DCR of 87% and a PFS of 5.6 months [13]. Additionally, preliminary results of a combined treatment with adagrasib and cetuximab demonstrated an ORR of 43% and a stable disease in 57% [13]. Further trials exploring the combination of anti-EGFR and anti-*KRAS* inhibitors are currently under clinical investigation with the aim of improving the efficacy of *KRAS* inhibitors [97].

### 2.4. BRAF^V600E^ Mutation

The RAS/MEK/ERK and PI3K pathways play a major role in cancer development and progression [98]. Among solid tumors, the prevalence of activating somatic missense *BRAF* mutations, with the *V600E* mutational variant accounting for about 80% of mutations, occurs in malignant melanomas (60–70%), papillary and anaplastic thyroid carcinomas (40–50%) and ovarian cancers (30%). All mutations in *BRAF* confer increased kinase activity compared with the wild-type protein and thereby stimulate *MAPK/ERK* activity in a *RAS*-independent manner. *BRAF* mutation occurs in about 10–20% of CRCs, and 90% of these mutations occur in *V600E*. *BRAF*-mutated CRCs patients show distinct clinico-pathological and molecular features identifying a subset with an aggressive phenotype and poor outcome [99]. More specifically, *BRAF*-mutated tumors are more frequently observed in elderly patients, females and in right-sided tumors, show more aggressive pathological features such as mucinous features, serrated architecture, poor differentiation, as well as peritoneal dissemination, and they present in an advanced stage at the time of the initial diagnosis [100]. Finally, the vast majority of patients with *BRAF*-mutated mCRC experience early disease progression [101]. 

*BRAF^V600E^* mutation is strongly associated with MSI-H/dMMR status (20–60%) [102]. Indeed, in sporadic CRCs, *BRAF* mutation is seen in approximately 60% of MSI-H tumors and only in 5–10% in MSS tumors [103]. This association is crucial also to distinguish sporadic MSI-H/dMMR from Lynch syndrome, since the *BRAF^V600E^* mutation excludes Lynch syndrome while being related to sporadic CRC patients [104].

The *BRAF^V600E^* mutation has been widely investigated, and its negative prognostic impact on stage II and III CRC has been observed in numerous studies [105,106]. Similarly, in advanced-stage patients with *BRAF* mutant CRC have low response rates to conventional therapies and poor OS [103].

Selective *BRAF* inhibitors are very effective as monotherapy in other malignancies, such as melanoma. However, disappointing results were observed in pretreated *BRAF* mutant mCRC patients with vemurafenib [107], suggesting that combination treatment could be more efficious to overcome primary resistance due to adaptive feedback, resulting in upstream activation of EGFR and subsequent treatment resistance [108]. 

The phase III BEACON trial was the first trial to show a substantial benefit both in survival and in response rates in pretreated *BRAF^V600E^* mCRCs [109]. This trial randomized 665 patients to receive triplet-therapy (encorafenib + binimetinib + cetuximab) or doublet-therapy (encorafenib + cetuximab) compared to the control arm (cetuximab + irinotecan or FOLFIRI). The results showed a clear advantage for the experimental arms: the doublet reported benefit in OS compared to control (9.3 months vs. 5.9 months, HR 0.61), in PFS and RR [110]. Results were almost identical with the triplet, yet with different and more frequent side effects. Based on these results, the doublet (encorafenib + cetuximab) was determined to be sufficient for OS and received FDA approval in April 2020. 

Of note, the results of this combination of drugs showed disappointing results in first-line *BRAF^V600E^* mCRC patients in the single-arm phase II trial (ANCHOR CRC) with a PFS of 4.9 months [111]. For this reason, the phase III BREAKWATER trial (NCT04607421) is currently ongoing and is comparing encorafenib + cetuximab alone or in combination with a chemotherapy doublet versus the doublet or triplet chemotherapy +/− bevacizumab in first-line *BRAF^V600E^* mutated mCRC [12].

Just recently, encouraging results of a phase I/II trial exploring the efficacy of encorafenib and cetuximab plus nivolumab in 26 patients with MSS/*BRAF^V600E^*-mutated mCRC were presented at the 2022 Gastrointestinal Cancers Symposium [112]. The ORR and DCR were 50% and 96%, and mPFS and mOS were 7.4 and 15.1 months, respectively. 

Based on data available and due to the strong association between MSI-H and *BRAF* mutations, we believe that patients with MSI-H/*BRAF^V600E^*-mutated mCRC should be treated with CPI in the first-line setting, followed by a combined targeted treatment consisting of encorafenib and cetuximab in case of disease progression. 

### 2.5. HER2 Amplification

Amplification in the *HER2/Erbb2* oncogene or overexpression of its protein produces a hyperactivation of mitogenic signals, leading to uncontrolled cell proliferation and tumorigenesis [113]. *HER2* amplification has been reported in about 2–5% of all CRCs [114].

The role of *HER2* amplification as a prognostic biomarker is still uncertain [115], while its role as a negative predictive factor of resistance to anti-EGFR agents is more and more established [116]. 

More importantly, *HER2* amplification is a well-known druggable target in other settings, such as in breast and gastric cancers, in which approved treatments are already available.

In mCRC, the proof-of-concept multicenter, open-label, phase II trial HERACLES reported promising results with the combination of trastuzumab and lapatinib in heavily pretreated mCRC harboring a *HER2* amplification [117], although in a small population (n = 27), with an ORR of 30%. On the other hand, the HERACLES-B did not reach its primary endpoint of ORR. The HERACLES-B was a single-arm, phase II trial, in patients with histologically confirmed *RAS/BRAF* wild-type and *HER2*+ mCRC refractory to standard treatments [118]. 

Two further international phase II studies investigated the role of pertuzumab and trastuzumab combination in heavily pretreated patients: the MyPathway trial [119] and the TRIUMPH trial [120], with similar promising results. 

The ongoing phase II MOUNTAINEER trial is exploring the efficacy of tucatinib and trastuzumab in treatment-resistant mCRC [121]. Trastuzumab–deruxtecan (T-DXd) is an antibody–drug conjugate consisting of a humanized anti-*HER2* monoclonal specifically targeting *HER2*, with the same amino acid sequence as trastuzumab, a cleavable tetrapeptide-based linker and a potent topoisomerase I inhibitor as the cytotoxic drug (payload), who demonstrated revolutionizing and astonishing results in different malignancies [122]. The DESTINY-CRC01 was an open-label, phase II trial investigating the efficacy of T-DXd single agent in pretreated *HER2*-overexpressing mCRC patients [123]. In the HER2-positive cohort (defined as IHC3+ or IHC2+ and in situ hybridization (ISH)-positive), the ORR was 45.3%, and the mOS was 15.5 months. In addition, T-DXd seems to overcome resistance to previous HER2-targeted treatment, as no differences in outcome could be observed between patients who received HER2-targeted treatment in earlier lines and those being anti-HER2 treatment naïve [124]. 

Based on these results, the multicenter, randomized, double-blind, 2-arm, parallel phase II study DESTINY-CRC02 (NCT04744831) is currently ongoing [125]. Of note, in this trial *KRAS* mutant mCRC patients will also be enrolled, while in previous trials only *RAS* wild-type tumor patients were recruited.

## 3. Beyond Classical Biomarkers in CRC

### 3.1. NTRK Fusions

The neurotrophic tropomyosin receptor kinase genes (*NTRK1*, *NTRK2*, *NTRK3*) encode for tropomyosin receptor kinase proteins (TRK1, TRK2, TRK3) that are involved in embryonal neural development and in cell homeostasis [126,127]. Chromosomal fusions involving *NTRK* (e.g., *ETV6-NTRK3*, *CD74-NRTK1*) have been detected in a subset of cancer patients [128,129]. After promising results of preclinical experiments [129], clinical trials using two TRK inhibitors have been conducted. In a phase I/II trial, the first-generation TRK inhibitor larotrectinib was analysed for efficacy in tumor patients harboring a *NTRK* fusion [130]. An impressive ORR of 75% and long-lasting responses were observed. Not surprisingly, larotrectinib has been approved for the treatment of *NTRK*-rearranged malignancies, irrespective of tumor histology [131]. Similarly, treatment with entrectinib, an inhibitor targeting *TRK*, *ROS1* and *ALK*, was associated with clinically meaningful and durable responses in *NTRK*-fusion-positive tumors [15]. To overcome secondary resistance mechanisms, further second-generation TRK inhibitors are currently under clinical evaluation (i.e., repotrectinib [132] and taletrectinib [133]). 

In CRC, *NTRK* fusions are detectable in <1% of CRCs [134]. Molecular profiling data revealed that *NTRK* fusions are associated with *APC* and *TP53* mutations and are mutually exclusive of *RAS*/*RAF* alterations [134]. Furthermore, *NTRK*-rearranged tumors occur more frequently in MSI-H/dMMR CRCs [135]. Four CRC patients were treated with entrectinib within a clinical trial. Of them, one patient (25%) showed a partial response [15]. The ongoing phase II basket trial (NAVIGATE) investigates the effect of larotrectinib in patients with *NTRK* fusion-positive tumors. Preliminary data demonstrated an ORR of 50% and an mOS of 29.4 months in 10 patients with refractory *NTRK* fusion-positive mCRC [28].

Although *NTRK* fusions occur only in an extremely small subset of CRCs (<1%) and limited data exist in terms of the clinical efficacy of *NTRK* inhibitors in these tumors, larotrectinib and entrectinib are possible therapeutic options for *NTRK* fusion CRCs. Further research is needed to clarify the biological function and role of *NTRK* in CRC, especially in the subgroup of MSI-H/dMMR, and how this might affect response rates on anti-TRK inhibitors. 

#### 3.1.1. *FGFR*

Only recently, gene alterations within the fibroblast-growth factor receptor (*FGFR*) family have gained attention, since several different targeted agents showed clinically meaningful activity [136]. FGFR signaling is involved in numerous different pathways, including the MAPK and mTOR pathway [137,138]. The FGFR family includes four members: *FGFR1*, *FGFR2*, *FGFR3* and *FGFR4*. Gene fusions, mutations, and amplifications of the FGFR gene have all been reported in gastrointestinal cancers. In CRC, mainly amplifications in *FGFR1* and *FGFR2* are observed with an incidence of approximately 4% and 3%, respectively [139]. While in other *FGFR*-altered tumors, including cholangiocarcinomas [140] and bladder cancers [141], specific *FGFR* inhibitors yielded impressive response, in CRC data on *FGFR1* and *FGFR2* amplifications as novel targets are limited. In a retrospective study, investigators found that three of three patients with a *FGFR1*-amplified CRC showed a partial response upon regorafenib therapy [139]. Only recently, the activity of the *FGFR1-4* inhibitor futibatinib was assessed in a clinical phase I trial including five CRC patients in which only a modest activity could be described [142].

#### 3.1.2. DNA Damage Repair Genes

DNA damage repair (DDR) genes are essential for preserving the function of cells [143]. Several genes are involved in maintaining the stability of the DDR system, including *BRCA1*, *BRCA2* and *PALB2* [144]. Deficiencies in DDR genes via homologous recombination (HR) can cause genetic instability leading to increased rates of somatic genetic alterations [145,146]. Not surprisingly, for decades researchers tried to target these respective genes. Recently, poly(ADP-ribose)polymerase (PARP) inhibitors have been described to induce synthetic lethality in *BRCA*-mutated cancer cells in vitro [147] and are now increasingly used in *BRCA*-mutated tumors, such as pancreatic cancer [148]. Alterations in *BRCA* and in other DDR genes are found in a significant subset of gastrointestinal cancers (15–20%) [149]. Moreover, mutated DDR genes are detectable in approximately 22% of CRCs [144]. While the results of a first clinical trial investigating the efficacy of a monotherapy with the PARP inhibitor olaparib were disappointing [150], combining a PARP inhibitor with classical cytotoxic regimens yielded promising response rates [151,152]. Further agents beyond PARP inhibitors are currently under clinical investigation in several cancers. Of them, inhibitors targeting *ATR*, *CHK1* and *WEE1* seem to be very promising [153]. 

Since DDR gene mutations are detectable in a significant subset of CRC and several targeted agents currently being tested in clinical trials, these mutations represent a clinical meaningful target and will change the diagnostic and therapeutic management of CRCs in the near future. 

#### 3.1.3. POLE

DNA polymerase epsilon (*POLE*) encodes for the catalytic subunit of the DNA polymerase epsilon, which is involved in DNA replication and repair [154]. Mutations in *POLE* are observed in approximately 1–2% of CRC [155,156]. Interestingly, similarly to MSI-H tumors, *POLE*-mutated CRCs are characterized by the high infiltration of CD8^+^ T- cells [157,158] and a high TMB [159]. For this reason, researchers investigated the efficacy of the CPI nivolumab in mCRC patients harboring a *POLE* mutation. Five out of seven patients recruited in this study yielded a clinical response upon nivolumab monotherapy [159]. In a phase II trial, another CPI (durvalumab) was tested in MSI-H/dMMR or *POLE*-mutated mCRCs. Three patients with a *POLE* mutation were included. The efficacy of durvalumab could be observed in one patient [160]. Thus, *POLE* might serve as a potential predictive biomarker for CPI in patients with a MSS mCRC [161]. However, more research and clinical data are still needed to confirm this hypothesis.

#### 3.1.4. RET

Rearranged-during-transfection (*RET*) fusions occur in a subset of cancer patients, particularly in thyroid [162] and lung cancers [163]. *RET* rearrangements are detectable in 0.2–1.6% of CRCs [164,165]. *RET*-fused CRCs are more commonly detected in right-sided primaries, are associated with MSI-H/dMMR and with non-mutated *RAS/RAF* status. Regarding prognosis, they are linked to a shorter survival [166]. Interestingly, a negative predictive role of *RET* fusions upon anti-EGFR inhibitors were noted [89,167]. 

Even if the incidence of *RET* fusions in CRCs are rare, several approved multi-tyrosine kinase inhibitors (i.e., vandetanib, lenvatinib, ponatinib) are available that inhibit this specific pathway [168,169,170]. Just recently, Subbiah et al. presented preliminary data from the ongoing phase I/II LIBRETTO-001 trial (NCT03157128) [171]. Patients with mCRC harboring *RET* fusions and treated with selpercatinib achieved an ORR of 44% [16]. Another phase I/II trial is currently ongoing aiming to investigate the efficacy of pralsetinib (NCT03037385) in *RET*-rearranged tumors [172].

#### 3.1.5. RSPO Fusions and RNF43 Mutations

WNT/β-catenin signaling represents the most important altered pathway in the tumorigenesis of CRCs mainly induced by mutations in *APC* [173,174]. Many efforts were made to therapeutically inhibit WNT/β-catenin signaling. However, until now all studies failed to show any efficacy [175]. Interestingly, a subset of patients with CRC, pancreatic cancer and small bowel cancer harbor mutations in *RNF43* [176] or harbor an *R*-*spondin* (*RSPO*) fusion [177]. These genetic alterations are observed in a significant subset of CRC (8%) [178] and are mutually exclusive of mutations in *APC*. Currently, several early clinical trials are aiming to test the efficacy of various therapeutic agents, including LRP5/6 inhibitors (NCT03604445) [179].

#### 3.1.6. Other Promising Biomarkers

There are several post hoc translational studies from phase III randomized trials, such as FIRE-3 and TRIBE, demonstrating that variations in the *RSPO2*, *MKNK1* or GC genes involved in the MAPK and WNT signaling pathways predict outcome in patients with mCRC treated with FOLFIRI and bevacizumab [174,180,181]. These translational studies are critical to identify potential biomarkers in mCRC patients enabling us to better tailor treatment. 

### 3.2. A Selection of Emerging Biomarkers in the TME

The TME consists of various cell types such as cytotoxic and regulatory T-cells (CTL, Tregs), helper (Th) cells, natural killer (NK) cells, B-cells, myeloid-derived suppressor cells (MDSC), tumor-associated macrophages (TAM), cancer-associated fibroblasts (CAF) and endothelial cells [182,183,184]. These cells interact closely with cancer cells by promoting anti- and pro-tumorigenic effects and thus play a crucial role in modulating tumor growth, invasion and progression [17]. Pagès et al. could demonstrate in a large cohort of stage I–III colon cancer patients that an increased infiltration of CD3+ and cytotoxic CD8+ T-cells (Immunoscore high) is associated with a lower risk of disease recurrence and longer overall survival compared to those patients whose tumors exhibit a decreased CD3+/CD8+ density Immunoscore low) independent of age, T or N stages and MSI status. These results could be confirmed in further validation sets [185]. Additionally, the clinical utility of the Immunoscore could also be shown in mCRC patients [186,187]. Therefore, we can conclude that the CD3+/CD8+ immune infiltration within the TME may serve as a prognostic biomarker. However, whether the Immunoscore is predictive of response to CPI in MSS mCRC patients has to be further explored in prospective clinical trials. Guinney et al. categorized CRC into four molecular subgroups and created the consensus molecular subtypes (CMS) based on gene expression profiles [188]. The CMS1 group (MSI-like) is distinguished by a certain gene expression pattern being representative of activated cytotoxic T-cells. Thus, the CMS1 subtype represents an immunogenic TME and is in general associated with a favorable outcome [189]. In contrast, the CMS4 subtype (mesenchymal) is characterized by a stroma-rich and immunosuppressive TME and mainly associated with a poor outcome [189]. While the CMS2 subtype (canonical) comprises CRC with predominantly activated WNT and Myc signaling pathways, the CMS3 subgroup (metabolic) is enriched by *KRAS* mutations and activated metabolic pathways. Both of them exhibit a decreased immune-related gene expression profile and are associated with an intermediate prognosis [189]. Although the prognostic impact of the CMS classification could have been shown and retrospective translational analysis of phase III clinical trials [190,191] demonstrated an added predictive value, its implementation into the daily diagnostic algorithm has not been established yet. This is mainly due to the labor-intensive, complex and costly analyses preventing a translation into the daily diagnostic workflow.

### 3.3. Circulating Tumor DNA (ctDNA)

Tumor cells shed ctDNA into the bloodstream. These ctDNA fragments possess the same genetic information as the primary tumor or the metastases [18]. The fraction of ctDNA is highly variable, ranging from <0.1% up to 10% depending on tumor load, tumor stage, tumor shedding and disease site [192,193]. In CRC, the use of ctDNA is not yet implemented in clinical practice. However, findings from recent studies and ongoing trials indicate that ctDNA may serve as both prognostic and predictive biomarkers in the near future. For a more comprehensive overview about ctDNA in colorectal cancer, we suggest further literature [194].

Briefly, ctDNA can be used with different aims in the adjuvant and metastatic setting. 

In early-stage patients ctDNA may be implemented at diagnosis for molecular profiling, especially if tumor tissue is not available. Moreover, ctDNA may be used as a biomarker for response to neoadjuvant and adjuvant therapies to detect minimal residual disease (MRD) after surgery and to guide treatment selection. For instance, the concentration of ctDNA in pretreatment plasma is significantly lower in stage I patients compared with stage II–III patients [195]. 

Recently, Tie et al. demonstrated that a ctDNA-guided treatment approach in stage II colon cancer patients reduced the use of adjuvant chemotherapy without compromising RFS [196]. Although other studies are needed, this study opens new avenues for the best selection of patients who are candidates for adjuvant treatment.

On the other hand, in the metastatic setting, ctDNA holds many promises in the clinic: molecular profiling at diagnosis, targeted therapy selection, treatment response monitoring, post-treatment tumor surveillance to detect recurrence, and monitoring treatment resistance [197,198]. 

Several trials are ongoing to investigate the clinical utility of ctDNA in both early-stage and advanced CRC patients, and the results derived from these studies will potentially change our clinical practice soon.

## 4. Preclinical Models for Target Discovery

### 4.1. Patient Derived Organoids

Given the high costs and the long timeline of new drug development, repurposing of already approved drugs used in other malignancies is a promising, cost-effective and efficient way to rapidly implement drugs with a known safety profile in the treatment algorithm of mCRC. A promising approach to simultaneously test multiple different single drugs or drug combinations and to assess their cytotoxic effect on tumor cells are patient-derived organoids (PDO) from patients with mCRC [199]. PDO are three-dimensional ex vivo cultured cancer cells that derive directly from the patient’s tumor. PDO reflect the in vivo heterogeneity and complexity of the tumor while retaining the accessibility of in vitro technologies. Preliminary studies could demonstrate that organoids exhibited similar molecular profiles as those observed in the patient’s tumor and may help unraveling resistance mechanisms to certain drugs during the course of the disease [200]. Additionally, there is evidence that PDO drug screens have the potential to predict therapeutic response in mCRC patients. Currently, several trials are ongoing to test organoid-based treatment prediction using already established standard of care regimens. However, the clinical utility of prospective PDO-based drug screening that goes beyond testing for standard of care options in patients with mCRC has not been largely explored yet. The APOLLO trial demonstrated that PDO-guided treatment might be promising to expand therapeutic options in patients with treatment-refractory peritoneal mCRC. However, the small number of patients included in this trial precludes drawing any definite conclusions [201]. An obvious limitation of classical PDO is that they do not mirror the complexity of the TME. However, further development of PDO and adding of co-cultures containing compounds of the extracellular matrix, such as endothelial cells, fibroblasts and immune cells, are highly warranted to accurately predict response to treatment that targets TME, including the immune system (e.g., CPI) [202]. Another issue is to obtain enough fresh tumor tissue for culturing, as sufficient tissue should also be available for routine diagnostic procedures at the same time. A further challenge in the clinical translation of PDO is to reduce the turnaround time, that means to minimize the time from obtaining tumor tissue for generating PDOs until the clinician receives the PDO drug screen results. In case of successful identification of potential effective compounds, further hurdles have to be overcome such as access to off-label drugs. Such a process involves a request for cost reimbursement from the health insurance company or the willingness of the pharmaceutic company to provide the drug for free. This approach is inevitably accompanied by the uncertainties of obtaining (or not) off-label compounds to treat such patients.

In conclusion, there is an unmet need to further explore the promising use of patient avatars such as PDO to move the field forward and to find new ways to optimize precision medicine and to individualize further-line treatment in mCRC patients. Further alternative models are described below.

### 4.2. Patient-Derived Xenograft Models (PDX)

Various murine models have been developed in the past to better understand tumorigenesis, tumor cell migration, invasion, metastasis and response to treatment [203]. A widely used model is the patient-derived xenograft (PDX), where human tumor cells are directly injected or transplanted into immunocompromised mice [203]. Whereas this model has the potential to examine response to therapy in vivo, several limitations do exist. First, the establishment and maintenance of PDX models is labor-intensive and associated with high costs. Second, tumor growth can last several months and may not guarantee efficient drug testing that can be rapidly translated into the real-life situation in the clinic, when the patient is faced with tumor progression and a further-line treatment has to be initiated within a reasonable time frame. Third, immunocompromised mice do not mirror the conditions in human cancers and may not serve as an optimal model to study response to CPI or interactions between the host’s immunity and the tumor. 

As our understanding of the significance of the TME and the host’s immune system is evolving, adapted models are eagerly needed to further advance in the field of precision medicine.

### 4.3. Patient-Derived Tumor Fragment Platform (PDTF)

Just recently, Voabil et al. developed an ex vivo tumor fragment culture that preserves the cellular composition and the architecture of the tumor. This platform maintains the characteristics of the TME and allows for example to study ex vivo CPI, mechanisms of resistance and various interactions between the tumor and its surrounding microenvironment. Moreover, Voabil et al. could demonstrate that there is a high correlation between clinical and ex vivo responses to CPI. This promising observation may lead us to further explore whether a PDTF platform helps to predict outcomes in cancer patients treated with CPI [204]. 

## 5. Challenges in Biomarker Research and Future Perspective

The dream of precision oncology is to individualize the diagnostic and therapeutic management of cancer patients according to their genetic profile. The increasing use of high-throughput techniques, such as next-generation sequencing (NGS), helped to pave the way for the implementation of precision oncology in our daily routine. The turn-around time of NGS results for established predictive biomarkers in the first-line setting (MSI, *RAS*, *BRAF*) could be significantly shortened and is currently about 5–7 days, enabling a timely start of biomarker-driven therapy. For these biomarkers, corresponding drugs are available and reimbursed by health insurance companies. Regarding the therapeutically relevant assessment of MSI/MMRd for first-line treatment, IHC testing for MMR status is even faster than NGS and allows a very early initiation of CPI in case of MMRd mCRC. The NGS results report not only contains informations on MSI, *RAS* and *BRAF* status but also includes comprehensive details on further druggable genetic alterations, such as *HER2* amplifications and *NTRK* fusions, that may be important in the further-line setting [117,131]. Although *HER2* amplification or overexpression of its protein is considered an established predictive biomarker in the further-line treatment of mCRC patients [117], anti-HER2 drugs are not yet approved in this setting and not reimbursed and only available within clinical trials or upon off-label request. Despite the low prevalence of *NTRK* fusions in mCRC [134], response rates to targeted treatment are encouraging [131,154], and drugs such as larotrectinib and entrectinib are available and reimbursed by the National Health System. However, while the clinical utility of single biomarkers, such as *KRAS* [205] and MSI-H/dMMR [10], have been proved to extend survival of CRC patients, the role of comprehensive molecular profiling remains largely questionable. Moreover, only a limited number of studies have investigated the cost-effectiveness of molecular profiling in cancer patients [206,207,208,209,210]. For instance, a study conducted in NSCLC patients showed that NGS-based parallel testing was more cost-effective than single-gene-based testing [207]. To the best of our knowledge, no similar study exists for CRC patients. For this reason, a health–economic analysis investigating the clinical utility, in terms of survival benefit, and the cost-effectiveness of comprehensive molecular profiling would be highly desirable. Additionally, the evidence of emerging biomarkers in patients with mCRC is mainly based on post-hoc, retrospective translational work and unplanned subgroup analysis from large clinical trials. Due to the low prevalence of potentially druggable oncogenic drivers in mCRC, biomarker-driven clinical studies focusing on evaluating targeted drugs in a single tumor entity are challenging, labor-intensive and costly. With the introduction of basket trials exploring new drugs that target a distinct genetic alteration irrespective of the type of cancer, these hurdles could be at least partly overcome [211]. 

Emerging biomarkers in the TME such as the Immunoscore or the CMS classification have proven their prognostic impact [185,188,189]. However, their predictive value in mCRC has still to be proven in prospective trials. One of the reasons why the CMS classification did not find its way into the clinic so far are the resource-intensive and costly analyses impeding its clinical implementation. PDX models or ex vivo platforms such as PDO or PDTF might be promising translational tools for biomarker development and drug testing in the near future. However, their role as patient avatars has to be further developed and optimized before an implementation into the daily clinical practice can be realized. 

With the introduction of new therapeutic approaches, the need for biomarkers will steadily increase in the future to identify subgroups of patients who may derive the most benefit from a specific treatment. While immunotherapies, targeted and cellular-based therapies have already found their way into the clinic for several malignancies, further innovative treatment approaches are emerging, such as cellular therapies, combined therapy using oncolytic viruses or genetically modified attenuated bacteria with immune-checkpoint inhibitors (NCT03256344, NCT05120596) [212,213]. Additionally, repurposing of drugs will be crucial to enlarge the treatment armamentarium against colorectal cancer. However, it is still debated whether ex vivo models such as PDX, PDO or PDTF may help us to accelerate this process.

In the future, to improve the selection of CRC patients for individualized therapies, we will need more informative profiling. However, we are still at the beginning of comprehensive molecular profiling, since we are still focusing (mostly) only on genetic alterations. Thus, to better depict the unique properties of cancer, we will need to include more information concerning the tumor’s biology. For instance, gene expression analyses might help to better characterize the link between genetic landscape and the cancer’s behavior [214,215]. Currently, understanding the complex interplay between CRC cells and the surrounding TME (e.g., immune cells, fibroblasts, healthy enterocytes and the gut microbiome) is a main focus in cancer research [216,217]. Findings from these analyses might help to establish a “real” comprehensive molecular profile that might serve as an imperative tool for precise therapies in CRC. 

Therefore, future development of precision medicine in metastatic colorectal cancer will not only focus on tumor cells but rather on implementing the tumor microenvironment in clinical decision-making. Notably, recent advances have elucidated that gut microbiota has different and complex roles in cancer development, as well as in treatment response, that must be comprehensively addressed from a clinically translational angle [218]. Various studies demonstrated that the composition of intestinal bacteria differs between responders and non-responders to immune checkpoint inhibitors [219] and also to chemotherapy. Several studies are ongoing to investigate the potential of utilizing gut microbiome as CRC biomarkers and also to enhance treatment efficacy and reduce adverse effects of CRC treatments [220].

Furthermore, combining information coming from genomics, transcriptomics, epigenomics, metabolomics, microbiomics and proteomics might help to establish a “real” comprehensive molecular profile that might serve as an imperative tool for precise therapies in CRC. 

Finally, artificial intelligence and digital pathology will help us to define nomograms integrating clinical, molecular and histopathological factors, while we are moving forward in the field of precision medicine.

## 6. Conclusions

A biomarker-driven treatment algorithm allows to identify those mCRC patients who derive the most benefit from a specific therapeutic approach, while we are further pursuing the path of personalized therapy.

## Figures and Tables

**Table 1:** Selection of requirements for an effective prognostic or predictive biomarker *.

Prediction with high accuracy [19,20]
Cost effective [19,21]
Easy to assess [20,21]
Reproducible/Reliable [19,21]
Consistent and rapid turnaround time [22]
Prognostic/predictive impact confirmed in validation sets [19,20,21]
Proven useful in a given clinical context [19,20]
Longitudinal and non-invasive measurements [23]

* This selection reflects the authors’ opinions and is not intended to be exhaustive.

**Table 2:** Evidence of established biomarkers in metastatic colorectal cancer patients in selected clinical trials.

Biomarker	Trial	Phase	N	Setting	Treatment	Outcomes
**Sidedness** (retrospective analyses, except PARADIGM)	CALGB/SWOG 80405	III	476	First-line mCRC	FOLFOX/FOLFIRI + cetuximab vs. FOLFOX/FOLFIRI + bevacizumab	mOS right 16.4 vs. 24.5 months mOS left 37.5 vs. 32.1 months
	FIRE-3	III	394	First-line mCRC	FOLFIRI + cetuximab vs. FOLFIRI + bevacizumab	mOS right 18.3 vs. 23.0 months mOS left 38.3 vs. 28.0 months
	PEAK	II	143	First-line mCRC	FOLFOX + panitumumab vs. FOLFOX + bevacizumab	mOS right 17.5 vs. 21.0 months mOS left 43.4 vs. 32.0 months
	PARADIGM	III	802	First-line mCRC	FOLFOX + panitumumab vs. FOLFOX + bevacizumab	mOS right 20.2 vs. 23.2 months mOS left 37.9 vs. 34.3 months
	CRYSTAL	III	364	First-line mCRC	FOLFIRI + cetuximab vs. FOLFIRI	mOS right 18.5 vs. 15.0 months mOS left 28.7 vs. 21.7 months
	PRIME	III	408	First-line mCRC	FOLFOX + panitumumab vs. FOLFOX	mOS right 11.1 vs. 15.4 months mOS left 30.3 vs. 23.6 months
**dMMR/MSI-H**	KEYNOTE-164	II	124	MSI-H/dMMR mCRC treated with ≥2 prior lines of standard therapy	Pembrolizumab	PFS 31% at 24 months OS 55% at 24 months
	CheckMate-142	II	74	MSI-H/dMMR mCRC who had ≥ 1 prior treatment	Nivolumab	PFS 36% at 48 months OS 49% at 48 months
	CheckMate-142	II	119	MSI-H/dMMR mCRC who had ≥1 prior treatment	Nivolumab 3 mg/kg + ipilimumab 1 mg/kg q3w × 4 followed by nivolumab 3 mg/kg q2w	PFS 53% at 48 months OS 71% at 48 months
	KEYNOTE-177	III	307	First-line MSI-H/dMMRmCRC	Pembrolizumab	mPFS 16.5 months mOS not reached
***RAS***	CRYSTAL	III	retrospective analysis of 827 pts evaluable for *RAS* mutational status	First-line mCRC	FOLFIRI vs. FOLFIRI + cetuximab	*RAS* wild-type mOS 20.2 vs. 28.4 months, HR 0.69 *RAS* mutant mOS 17.7 vs. 16.4 months, HR 1.05
	PRIME	III	656	First-line mCRC *KRAS* wild-type	FOLFOX vs. FOLFOX + panitumumab	mOS 19.4 vs. 23.8 months, HR 0.83
	PARADIGM	III	604	First-line mCRC *RAS* wild-type left-sided	FOLFOX + bevacizumab vs. FOLFOX + panitumumab	mOS 34.3 vs. 37.9 months, HR 0.82
***KRAS^G12C^***	CodeBreak 100	II	62	Refractory mCRC	Sotorasib	ORR 9.7%, DCR 82.3%
	KRYSTAL-1	I/II	46	Refractory mCRC	Adagrasib	ORR 22%, DCR 87%
***BRAF^V600E^***	BEACON	III	665	Refractory mCRC	Encorafenib + binimetinib + cetuximab vs. encorafenib + cetuximab vs. FOLFIRI or cetuximab + irinotecan	mPFS 4.5 vs. 4.3 vs. 1.5 months mOS 9.3 vs. 9.3 vs. 5.9 months
***HER2***	HERACLES	II	27	Refractory mCRC	Lapatinib + trastuzumab	ORR 30%
	HERACLES-B	II	31	Refractory mCRC	Pertuzumab + trastuzumab-emtansine	ORR 9.7%
	MyPathway	II	43	Refractory mCRC *KRAS* wild-type	Pertuzumab + trastuzumab	mPFS 5.3 months mOS 14.0 months ORR 40%
	TRIUMPH	II	27	Refractory mCRC	Pertuzumab + trastuzumab	mPFS 4.0 months mOS 10.1 months ORR 30%
	MOUNTAINEER	II	26	Refractory mCRC	Tucatinib + trastuzumab	mPFS 6.2 months mOS 17.3 months ORR 55%
	DESTINY-CRC01	II	53	Refractory mCRC	Trastuzumab-deruxtecan	mPFS 6.9 months mOS 15.5 months ORR 45.3%
***NTRK***	NAVIGATE	II	10	Refractory mCRC *NTRK* fusion-positive	Larotrectinib	mPFS 5.5 months mOS 29.4 months ORR 50%
	ALKA-372-001, STARTRK-1, and STARTRK-2	I/II	4	mCRC *NTRK* fusion-positive	Entrectinib	ORR 25%

DCR, disease control rate; FOLFIRI, 5-fluorouracil + irinotecan; FOLFOX, 5-fluorouracil + oxaliplatin; dMMR, DNA mismatch repair deficiency; mCRC, metastatic colorectal cancer; MSI-H, microsatellite instability high; OS, overall survival; ORR, objective response rate; PFS, progression-free survival.
